# PyToxo: a Python tool for calculating penetrance tables of high-order epistasis models

**DOI:** 10.1186/s12859-022-04645-7

**Published:** 2022-04-02

**Authors:** Borja González-Seoane, Christian Ponte-Fernández, Jorge González-Domínguez, María J. Martín

**Affiliations:** 1 Facultad de Informática, Universidade da Coruña, 15071 A Coruña, Spain; 2 CITIC, Computer Architecture Group, Facultad de Informática, Universidade da Coruña, 15071 A Coruña, Spain

**Keywords:** Simulation, Epistasis model, Gene interaction, Penetrance, Prevalence, Heritability, Python, SymPy

## Abstract

**Background:**

Epistasis is the interaction between different genes when expressing a certain phenotype. If epistasis involves more than two loci it is called high-order epistasis. High-order epistasis is an area under active research because it could be the cause of many complex traits. The most common way to specify an epistasis interaction is through a penetrance table.

**Results:**

This paper presents PyToxo, a Python tool for generating penetrance tables from any-order epistasis models. Unlike other tools available in the bibliography, PyToxo is able to work with high-order models and realistic penetrance and heritability values, achieving high-precision results in a short time. In addition, PyToxo is distributed as open-source software and includes several interfaces to ease its use.

**Conclusions:**

PyToxo provides the scientific community with a useful tool to evaluate algorithms and methods that can detect high-order epistasis to continue advancing in the discovery of the causes behind complex diseases.

**Supplementary Information:**

The online version contains supplementary material available at 10.1186/s12859-022-04645-7.

## Background

Genome-Wide Association Studies (GWAS) analyse genetic markers to find associations between genetic variations and diseases. The genetic markers commonly used in GWAS are the Single Nucleotide Polymorphisms (SNP). A SNP identifies a specific position (locus) in the genome where at least 1% of the population has a genomic variation.

Traditional GWAS focused on analysing the differences between the genotype frequencies from individual SNPs of case and control samples. However, epistasis interactions need to be considered to find a relation between genotypes and phenotypes in many traits [1].

Epistasis is the interaction of genetic variation at two or more loci to produce a phenotype that is not explained by the additive combination of effects of the individual loci. If epistasis involves more than two loci, it is called high-order epistasis. High-order epistasis is behind complex diseases such as Alzheimer’s [2] or breast cancer [3].

The most common way to describe an epistasis relationship is through a penetrance table. A penetrance table captures the probability of expressing the phenotype to study given a particular allele combination. Table 1 shows an example of a penetrance table that considers the effects of two biallelic SNPs.

**Table 1:** Example of a penetrance table for two biallelic markers

	BB	Bb	bb
AA	0.00031	0.00031	0.00031
Aa	0.00031	0.00231	0.01750
aa	0.00031	0.01750	1

In the literature, there are simulators, such as EpiSIM [4], that allow obtaining a penetrance table for a previously established prevalence (*P*(*D*)) and heritability ($h^2$). These penetrance tables are obtained through the solution of the following system of equations:

1 $$\begin{aligned} P(D)= & {} {\sum _i} P(D|g_i) P(g_i) \\ h^2= & {} \frac{ {\sum _i} \big (P(D|g_i) - P(D)\big )^{2} P(g_i)}{P(D) \big (1 - P(D)\big )} \end{aligned}$$

where $P(D|g_i) = f_i(x,y)$ is the proportion of individuals showing trait *D* when having the genotype $g_i$, $P(g_i)$ is the population frequency of the genotype $g_i$ and $f_i(x,y)$ is the function of two variables that defines the epistasis model. Table 2 shows an example of this function for the second-order Marchini et al. [5] additive model.

However, as not all combinations of heritability and prevalence are possible, the system often is incompatible. Additionally, equations become more and more complex as the interaction order increases, which also makes it difficult to find a solution in a reasonable time. Thus, for example, the EpiSIM simulator can only work, in practice, with second-order models and low prevalence and heritability values [6].

**Table 2:** Second-order additive model from [5]

	BB	Bb	bb
AA	*x*	$x (1 + y)$	$x (1 + y)^2$
Aa	$x (1 + y)$	$x (1 + y)^2$	$x (1 + y)^3$
aa	$x (1 + y)^2$	$x (1 + y)^3$	$x (1 + y)^4$

To overcome these limitations, in a previous work we introduced Toxo [6], a MATLAB library for calculating penetrance tables of epistasis models with no limitation on the interaction order. To simplify the equation system shown in Eq. 1, instead of finding a specific combination of heritability and prevalence, the Toxo library maximizes one of the two parameters when the other is fixed. The system to solve for a given value of prevalence is shown in Eq. 2:

2 $$\begin{aligned}{\sum_{i}} \big(P(D|g_i) P(g_i)\big )&= P(D) \\max\big (P(D|g_i)\big )&= 1 \end{aligned}$$

Equation 3 shows the system for a fixed heritability:

3 $$\begin{aligned}\frac{ {\sum_{i}}\big (P(D|g_{i}) - P(D)\big )^2 P(g_{i})}{P(D)\big (1 - P(D)\big )}&= h^{2} \\max\big (P(D|g_{i})\big )&= 1 \end{aligned}$$

Now, the likelihood of formulating an incompatible system is significantly reduced, since most of the models achieve individually all prevalences and heritabilities values at some point. Moreover, Toxo can calculate penetrance tables with prevalence and heritability values much higher than those observed in the state of the art. That is, Toxo provides researchers with a library that is able to generate more realistic penetrance tables.

Although Toxo solves several of the shortcomings of state-of-the-art simulators, it also has its own limitations. First, MATLAB is a commercial software and the user will need a license to run Toxo. In addition, Toxo is a library and thus, it requires certain programming knowledge to use it. It also presents limitations in the accuracy of the results, motivated by the compromise between computing time and precision that users are forced to make in MATLAB when selecting the number of decimals to operate with variable-precision arithmetic.

To solve all these limitations, in this work we present PyToxo, a Python tool to calculate penetrance tables for high-order epistasis models. PyToxo is distributed as open-source software and it does not need any commercial license since Python is open-source itself. In addition, as we will see in the results section, PyToxo improves Toxo in terms of the complexity of the epistasis models that it can handle, the accuracy in the obtained penetrance table and the execution time needed. Finally, regarding the ease of use, PyToxo includes a programmatic interface, in the form of a library, to be easily used in other Python programs; a Command-Line Interface (CLI) for advanced users, or to be able to execute the program in batch processing mode; and a Graphical User Interface (GUI) specially oriented for users unfamiliar with command-line runtime environments.

## Implementation

PyToxo is implemented in Python. Currently, Python is one of the most widely used programming languages [7, 8] thanks to its simple and high-level syntax, and the large number of available libraries. In fact, it is one of the most frequently used options in the interdisciplinary field of bioinformatics, as evidenced by the Biopython project [9].

PyToxo uses multiple Python libraries in its implementation. The most important is *SymPy* [10, 11], a symbolic math library used to solve the equation systems presented in Eqs. 2 and 3, and to represent the numerical data. Additionally, PyToxo also uses *mpmath* [12] to tweak the precision of the floating-point arithmetic in *SymPy*, and *Pillow*, *PySimpleGUI* [13], *Termcolor* [14] and *Colorama* [15] to implement the different user interfaces.

### Method

**Fig. 1:**
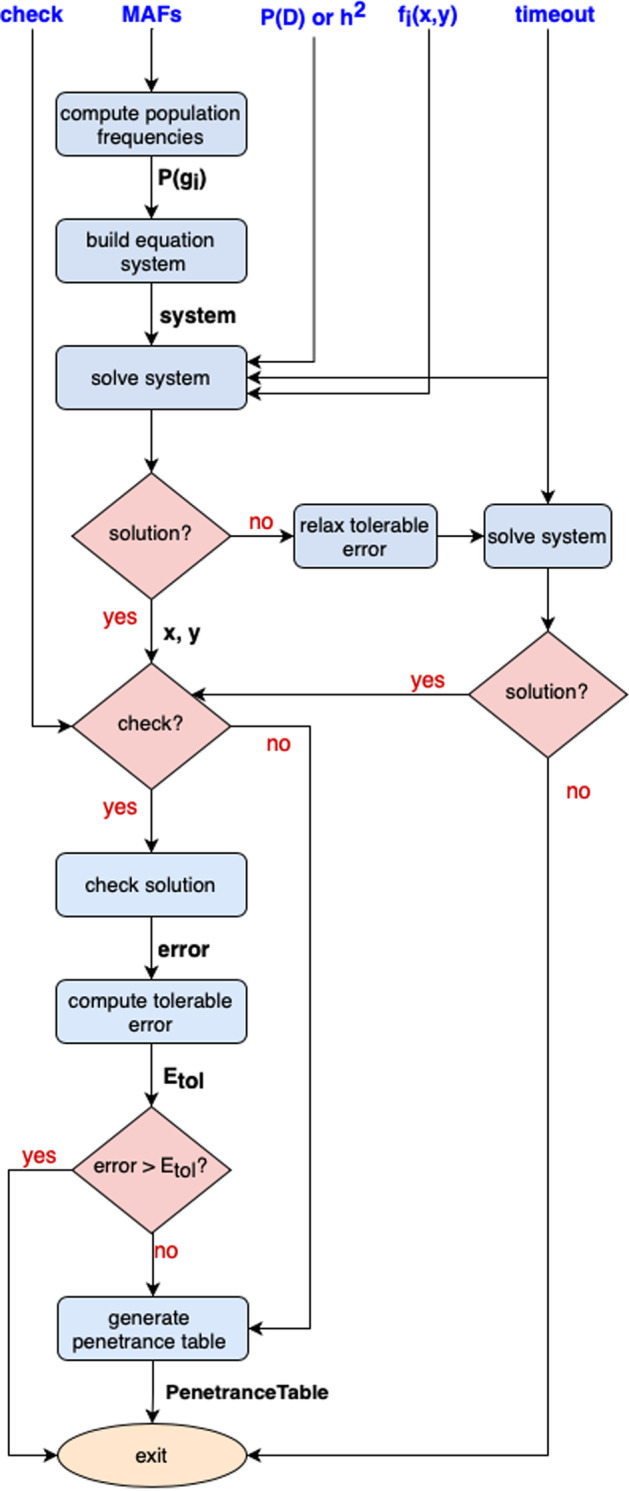
Generation of a penetrance table. Flowchart of the program

PyToxo takes as input an epistasis model, a heritability (or prevalence) value and the Minor Allele Frequencies (MAFs) associated to each of the considered locus, and generates as output a penetrance table maximizing the prevalence (or heritability). Figure 1 shows an overview of the flow of the program. $f_i(x,y)$ is the function of two variables that defines the epistasis model, *timeout* is the maximum time that the solver can spend trying to resolve the system, and *check* is a boolean variable that enables checking the correctness of the provided solution.

The first step consists of computing, from the MAFs, the population frequency associated to each of the genotype combinations, $P(g_i)$. Assuming linkage equilibrium between the two locus, and under the Hardy-Weinberg principle, $P(g_i)$ can be calculated as the product of the probabilities of each allele [16].

Then, the equation system to be solved is built using Eq. 2 for a fixed prevalence or Eq. 3 for a fixed heritability, and taking into account that $P(D|g_i) = f_i(x,y)$. The simplify() method of the *SymPy* library is used to simplify the mathematical expression of the system. It uses heuristics to determine the simplest result.

Next, the function solve() [17], from the *SymPy* library, is used to solve the resulting nonlinear system and obtain the values of *x* and *y*. It internally uses the *mpmath* library to control the accumulated error that is considered tolerable. In the case of PyToxo, especially when working with large models, it is possible to relax this accumulative error to try to converge to a solution of the system. Thus, when using the solve() function, an initial attempt is made to solve the system of equations without altering any configuration and, only if it is not possible, the accumulative error is increased and tried again.

Note that the solver could not converge for some configurations. Thus, to avoid deadlocks, a maximum time to achieve a solution is introduced through the timeout argument. If the user sets it to false, no maximum time will be established; if an integer is introduced, it will be interpreted as the maximum time, in seconds; and if it is set to true, the maximum time will be assumed heuristically taking into account the order of the model: $timeout = 60(order + 1)^2$.

Once a solution to the system of equations has been found, PyToxo can verify the precision of this solution if instructed to do so through the check variable. To do so, the error of the solution is computed as the deviation obtained when substituting the values of *x* and *y* calculated in the previous step into Eq. 2, if the prevalence is fixed, or into Eq. 3, when the fixed parameter is the heritability. PyToxo uses a heuristic function to calculate, based on the order, the tolerable error: $E{_{tol}(order)} = \min {(10^{order}E_{0}, E_{max})}$. In this function, $E_0$ represents the base error which scales with the order of the model and is set to ${10^{-16}}$, and $E_{max}$ is the maximum allowed error and is set to ${10^{-8}}$. If the obtained error is greater than the tolerable error, the solution is considered invalid and a warning is generated. Thus, the solutions provided by PyToxo will have a guaranteed maximum deviation of ${10^{-8}}$. In any case, PyToxo rarely shows deviations greater than ${10^{-16}}$, as we will see in “Results and discussion” section.

**Table 3:** Input parameters of PyToxo

Parameter	Description	Compulsory	Default value
*P*(*D*) (or $h^2$)	Established prevalence (or heritability)	Yes	–
MAFs	Minor allele frequencies of each locus	Yes	–
$f_i(x,y)$	Function of two variables that defines the epistasis model	Yes	–
check	Boolean variable that enables checking the correctness of the solution	No	True
Timeout	Maximum time to achieve a solution	No	True*

*Time is computed based on the order of the model

At last, once the values of *x* and *y* have been established, they can be directly used in the penetrance expressions $f_i(x,y)$ to obtain the target penetrance table. The generated table can be printed on the screen or saved to a file. For both cases, the supported formats are a Comma-Separated Values (CSV) file and the format used by the GAMETES [18] simulator. With the latter, the tables obtained by PyToxo can be passed directly to it in order to simulate population data that shows the epistasis interaction described by the penetrance table provided. As a summary, Table 3 shows a description of the parameters used by PyToxo together with their default values.

**Fig. 2:**
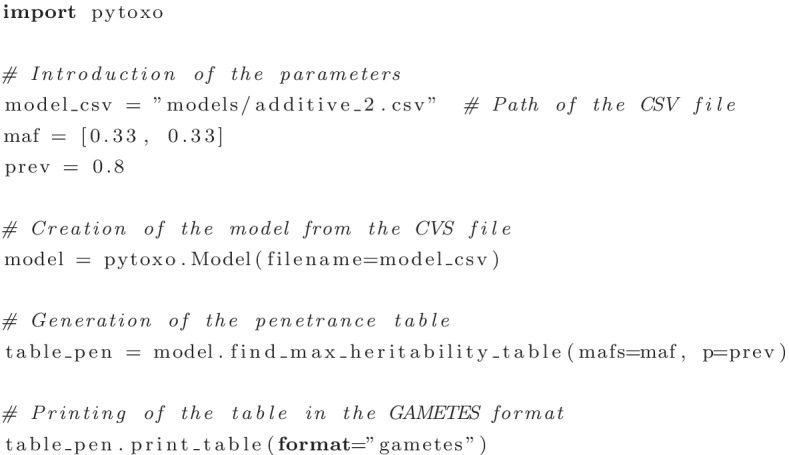
Use of PyToxo as a library in a Python program

### Interfaces

In addition to offer an API for developers (see Fig. 2 for an example of use), Pytoxo includes two user interfaces: a CLI for advanced users or to be able to run the program in batch processing; and a GUI, especially oriented for users unfamiliar with command-line execution environments.

#### Command-Line Interface (CLI)

For the implementation of the CLI, the *Argparse* library [19] was used. This library is included in the Python serial distribution and constitutes the official recommendation of the Python developers to build command-line interfaces. It facilitates the creation of CLIs, generating help messages, and ensuring the correct use of the parameters by the end user.

**Fig. 3:**
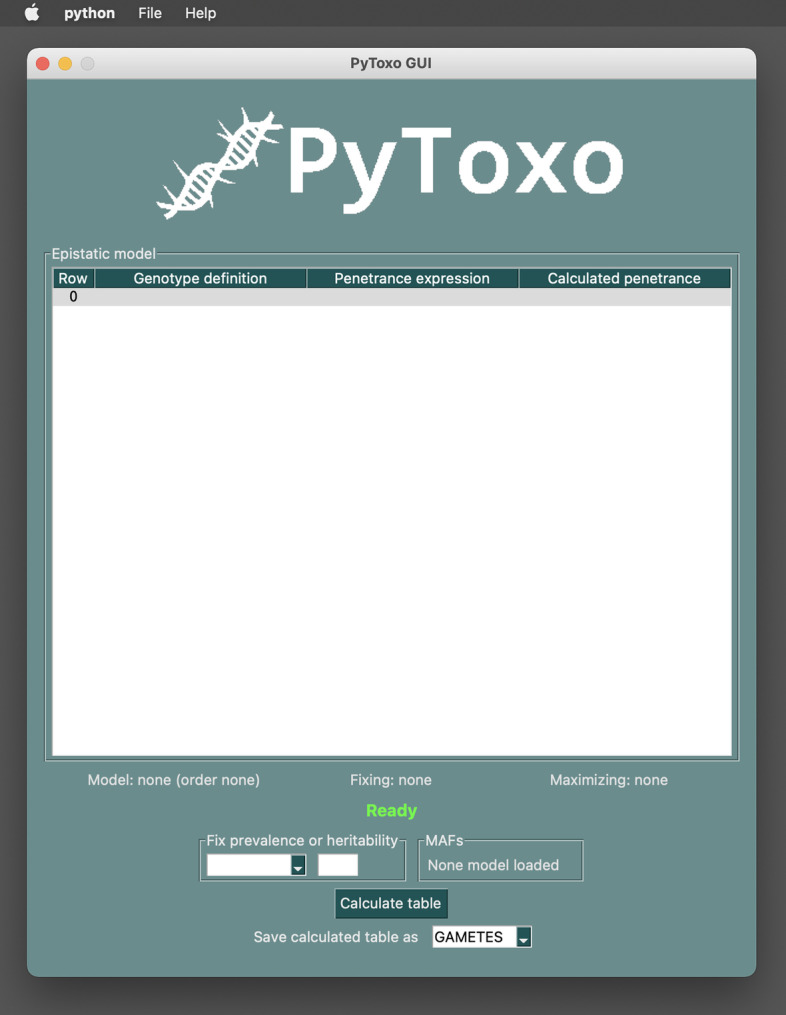
PyToxo GUI initial screen

The CLI allows calculating penetrance tables in both the GAMETES format and CSV. It accepts as input a CSV file containing the epistasis model to be used during the process, as well as the specification of the rest of the numerical parameters: MAFs, and prevalence or heritability, depending on which parameter is maximized. For instance, the next command would calculate a penetrance table for the fourth order Marchini et al. [5] multiplicative model, using a prevalence of 0.6 and MAFs of 0.2, 0.3, 0.3 and 0.4. Heritability is maximized and the output penetrance table is generated in GAMETES format:

pytoxo_cli multiplicative_4.csv –-max_her 0.6 0.2 0.3 0.3 0.4 –-gametes

#### Graphical User Interface (GUI)

The PyToxo GUI is built on *Tk*. *Tk* is a library of basic elements to build graphical user interfaces. In Python, *Tk* is accessed from the *Tkinter* [20] binding, and is considered a standard for graphical interfaces in Python. *Tk* has been ported to work on most variants of Linux, Apple Mac OS, and Microsoft Windows. Both *Tk* and *Tkinter* are included in most modern Python distributions for the above platforms. This is not the case for some Linux distributions, where an additional package must be installed. However, PyToxo itself detects this lack when trying to launch the interface and suggests to the user to install the required package. Thus, the graphical interface of PyToxo is not only highly compatible but also highly distributable, not needing any requirements outside of Python.

Although PyToxo’s graphical interface engine is *Tk*, PyToxo does not use it directly through *Tkinter* but uses a third library, *PySimpleGUI*, which simplifies the development of the interface. In addition, *PySimpleGUI* would allow changing the underlying *Tk* engine to another, such as *Qt* [21], with hardly any adjustments to the code, so the interface is also enhanced in terms of portability.

Figure 3 shows the initial screen of the GUI. The interface is very easy to use because it dynamically adapts to the workflow of the application. For example, we cannot use the “Calculate table” button until all the parameters have been filled in, and we cannot fill in the MAFs until we have loaded a model. The file menu is used to select the CSV file with the model.

## Results and discussion

This section evaluates Pytoxo in terms of coverage, accuracy and execution time, comparing it to Toxo.

### Coverage

In this first subsection we will address the coverage of PyToxo, that is, the models and configurations that it is able to solve.

**Table 4:** Some configurations, maximizing prevalence, that Toxo is not able to solve and PyToxo can

Model	Order	MAF	$h^2$
Additive	4	0.2	0.8
Additive	5	0.1	0.9
Additive	5	0.2	0.9
Additive	5	0.3	0.4
Additive	5	0.3	0.6
Additive	5	0.4	0.7
Additive	5	0.5	0.6
Additive	6	0.1	0.8
Additive	6	0.4	0.3
Additive	6	0.5	0.6
Additive	7	0.4	0.7
Additive	7	0.5	0.3
Additive	8	0.1	0.6
Additive	8	0.1	0.7
Additive	8	0.2	0.1
Additive	8	0.2	0.1
Additive	8	0.2	0.3
Additive	8	0.2	0.4
Additive	8	0.4	0.1
Additive	8	0.4	0.5

**Table 5:** Some configurations, maximizing heritability, that Toxo is not able to solve and PyToxo can

Model	Order	MAF	*P*
Multiplicative	6	0.10	0.30
Multiplicative	6	0.10	0.60
Multiplicative	6	0.10	0.80
Multiplicative	6	0.10	0.90
Multiplicative	6	0.20	0.30
Multiplicative	6	0.20	0.40
Multiplicative	6	0.20	0.50
Multiplicative	6	0.20	0.90
Multiplicative	6	0.40	0.20
Multiplicative	6	0.40	0.30

To evaluate PyToxo we select the widely used threshold, additive and multiplicative models introduced by Marchini et al. in [5] and generalized up to order 8. As for the rest of parameters, we considered a representative range of values: [0.1, 0.5] for the MAFs associated with each locus, and [0.1, 0.9] for both the heritability and prevalence. In total, we based our coverage experiments on 1890 different configurations, with half maximizing prevalence and half maximizing heritability. The results can be summarized as:

- PyToxo manages to solve all the configurations that Toxo solves except one.
- PyToxo manages to solve 201 configurations that Toxo is not able to solve.
- PyToxo is able to solve threshold and additive models up to, at least, order 8.
- PyToxo is able to solve multiplicative models up to order 5.

Tables 4 and 5 present a sample of particular configurations that PyToxo manages to solve, while Toxo ends up with corrupt tables. The results of all the performed experiments are available in the reports directory of the test.solubility package of the PyToxo repository (https://github.com/bglezseoane/pytoxo).

The limits of the tool appear with the multiplicative model, which is the most complex. Up to order 5, PyToxo performs well, but from that point on, most of the configurations are unsolvable.

### Accuracy

The accuracy of PyToxo depends on the order of the model. The error is calculated as the deviation between the obtained values of prevalence (or heritability) and the required ones. The higher the order, the greater the error, because the system of equations to be solved becomes more complex.

**Table 6:** Accuracy and execution time (in seconds) of Toxo and PyToxo for threshold, additive and multiplicative models with orders between 2 and 8 and different values of MAFs and heritability ($h^2$)

Model	Order	MAF	*h*^2^	PyToxo error	Toxo error	Best	PyToxo Time	Toxo Time	Speedup
Threshold	2	0.1	0.1	2.78$\times 10^{-17}$	2.45$\times 10^{-8}$	PyToxo	0.20	0.55	2.70
Threshold	2	0.1	0.8	0	0	Same	0.18	0.23	1.22
Threshold	2	0.4	0.1	1.11$\times 10^{-16}$	0	Toxo	0.14	0.24	1.70
Threshold	2	0.4	0.8	1.11$\times 10^{-16}$	0	Toxo	0.14	0.22	1.57
Threshold	3	0.1	0.1	1.39$\times 10^{-17}$	1.70$\times 10^{-7}$	PyToxo	0.39	0.31	0.79
Threshold	3	0.1	0.8	0	3.44$\times 10^{-9}$	PyToxo	0.37	0.71	1.91
Threshold	3	0.4	0.1	0	0	Same	0.31	0.30	0.98
Threshold	3	0.4	0.8	1.11$\times 10^{-16}$	0	Toxo	0.32	0.29	0.92
Threshold	4	0.1	0.1	0	7.41$\times 10^{-7}$	PyToxo	0.81	0.52	0.65
Threshold	4	0.1	0.8	0	1.60$\times 10^{-8}$	PyToxo	0.80	0.92	1.15
Threshold	4	0.4	0.1	2.78$\times 10^{-17}$	0	Toxo	0.67	0.52	0.77
Threshold	4	0.4	0.8	0	0	Same	0.67	0.53	0.77
Threshold	5	0.1	0.1	0	1.41$\times 10^{-6}$	PyToxo	1.95	1.99	1.02
Threshold	5	0.1	0.8	1.11$\times 10^{-16}$	5.71$\times 10^{-8}$	PyToxo	2.04	2.34	1.15
Threshold	5	0.4	0.1	1.39$\times 10^{-17}$	0	Toxo	2.39	1.98	0.83
Threshold	5	0.4	0.8	1.11$\times 10^{-16}$	4.17$\times 10^{-9}$	PyToxo	2.43	1.94	0.80
Threshold	6	0.1	0.1	1.39$\times 10^{-17}$	1.95$\times 10^{-5}$	PyToxo	4.70	4.31	0.92
Threshold	6	0.1	0.8	2.22$\times 10^{-16}$	5.43$\times 10^{-8}$	PyToxo	4.68	4.26	0.91
Threshold	6	0.4	0.1	1.39$\times 10^{-17}$	0	Toxo	6.64	4.29	0.65
Threshold	6	0.4	0.8	0	5.05$\times 10^{-9}$	PyToxo	6.59	4.70	0.71
Threshold	7	0.1	0.1	1.39$\times 10^{-17}$	3.81$\times 10^{-5}$	PyToxo	11.69	18.27	1.56
Threshold	7	0.1	0.8	1.11$\times 10^{-16}$	1.58$\times 10^{-8}$	PyToxo	11.62	18.68	1.61
Threshold	7	0.4	0.1	4.16$\times 10^{-17}$	0	Toxo	18.50	18.33	0.99
Threshold	7	0.4	0.8	2.22$\times 10^{-16}$	0	Toxo	18.49	18.70	1.01
Threshold	8	0.1	0.1	2.78$\times 10^{-17}$	$2.42\times 10^{-4}$	PyToxo	28.72	39.03	1.35
Threshold	8	0.1	0.8	1.11$\times 10^{-16}$	1.29$\times 10^{-6}$	PyToxo	28.70	39.23	1.37
Threshold	8	0.4	0.1	1.39$\times 10^{-17}$	3.51$\times 10^{-8}$	PyToxo	52.58	38.91	0.74
Threshold	8	0.4	0.8	1.11$\times 10^{-16}$	0	Toxo	52.69	38.88	0.74
Additive	3	0.1	0.1	1.39$\times 10^{-17}$	1.05$\times 10^{-6}$	PyToxo	2.13	3.04	1.43
Additive	3	0.1	0.8	2.22$\times 10^{-16}$	3.26$\times 10^{-5}$	PyToxo	1.90	1.67	0.88
Additive	3	0.4	0.1	9.71$\times 10^{-17}$	0	Toxo	1.81	1.53	0.85
Additive	3	0.4	0.8	1.11$\times 10^{-16}$	1.62$\times 10^{-7}$	PyToxo	1.76	1.62	0.92
Additive	4	0.1	0.1	2.78$\times 10^{-17}$	5.54$\times 10^{-4}$	PyToxo	3.04	8.72	2.87
Additive	4	0.4	0.1	1.39$\times 10^{-17}$	0	Toxo	3.16	4.94	1.56
Additive	4	0.4	0.8	0	0	PyToxo	3.20	4.72	1.47
Additive	5	0.4	0.1	1.39$\times 10^{-17}$	2.66$\times 10^{-8}$	PyToxo	6.14	8.80	1.43
Additive	6	0.4	0.1	1.39$\times 10^{-17}$	6.37$\times 10^{-7}$	PyToxo	13.19	22.60	1.71
Additive	7	0.4	0.1	0	4.69$\times 10^{-7}$	PyToxo	25.86	51.82	2.00
Multiplicative	2	0.1	0.1	0	4.16$\times 10^{-6}$	PyToxo	0.67	2.84	4.25
Multiplicative	2	0.1	0.8	0	1.20$\times 10^{-7}$	PyToxo	0.69	1.01	1.47
Multiplicative	2	0.4	0.1	0	0	Same	0.71	1.01	1.43
Multiplicative	2	0.4	0.8	0	4.88$\times 10^{-9}$	PyToxo	0.64	1.00	1.56
Multiplicative	3	0.1	0.1	1.25$\times 10^{-16}$	7.51$\times 10^{-4}$	PyToxo	1.62	5.88	3.62
Multiplicative	3	0.1	0.8	0	3.59$\times 10^{-6}$	PyToxo	1.62	3.95	2.44
Multiplicative	3	0.4	0.1	4.16$\times 10^{-17}$	1.63$\times 10^{-7}$	PyToxo	1.66	2.96	1.78
Multiplicative	3	0.4	0.8	1.11$\times 10^{-16}$	1.39$\times 10^{-8}$	PyToxo	1.59	3.48	2.18
Multiplicative	4	0.1	0.1	8.33$\times 10^{-17}$	3.29$\times 10^{-17}$	PyToxo	4.69	83.18	17.75
Multiplicative	4	0.1	0.8	3.33$\times 10^{-16}$	3.57$\times 10^{-5}$	PyToxo	4.34	81.94	18.88
Multiplicative	4	0.4	0.1	1.39$\times 10^{-17}$	1.08$\times 10^{-6}$	PyToxo	4.55	54.42	11.94
Multiplicative	4	0.4	0.8	1.11$\times 10^{-16}$	3.60$\times 10^{-8}$	PyToxo	4.77	51.33	10.75

Table 6 shows the error of Toxo and PyToxo for the threshold, additive and multiplicative models and orders between two and eight. For each model and order, we have considered MAFs values of 0.1 and 0.4, and heritabilities of 0.1 and 0.8 for each MAF. Results with more MAFs, heritabilities and prevalences values have been added as Additional file 1. The configurations not shown in the table correspond to combinations where either Toxo or PyToxo did not achieve valid solutions. PyToxo obtains a lower (or equal) error than Toxo in 39 out of the 50 cases shown in the table. Additionally, the average error in PyToxo is ${5.74\times 10^{-17}}$ versus $1.78 \times 10^{-4}$ in Toxo, and in the few cases where Toxo obtains better accuracy results, the error introduced by PyToxo is also negligible (in the order of ${10^{-16}}$ or ${10^{-17}}$).

With all of this in mind, we can conclude that PyToxo significantly improves the accuracy of the solutions.

### Execution time

This section compares the execution times of Toxo and PyToxo for the same configurations as the previous section. Results with more MAF, heritability and prevalence values are available as Additional file 1. All the tests were executed on an Intel Core i5-9600K with 6 cores at 3.7 GHz, 16 GB of RAM DDR4, and Operating System Mac OS Big Sur 11.2.1. The MATLAB version used to execute Toxo has been the R2020b. Table 6 shows the execution times using Toxo and Pytoxo and the speedup obtained with PyToxo.

The time needed by PyToxo to calculate a penetrance table grows with the order of the model to use. However, for the models tested, PyToxo is very fast, taking in all cases less than a minute.

With respect to Toxo, PyToxo obtains an average speedup of 1.90. However, if we focus on the most complex and time-consuming models, such as the fourth-order multiplicative models, the speedup of PyToxo over Toxo increases above 10.

In view of the results obtained, PyToxo would become the tool of choice for obtaining penetrance tables, regardless of the epistatic model and input parameters considered.

## Conclusions

The best way to test new algorithms or methods to detect high-order epistasis is through simulated data since they provide a controlled environment where the embedded epistatic interactions are known in advance.

Although it is very common for simulators to use penetrance tables to describe epistasis interactions, most of them do not allow the user to generate them directly, or present limitations for high-order interactions and/or realistic prevalence and heritability values.

PyToxo is a Python tool to calculate penetrance tables for high-order epistasis models. It improves state-of-the-art tools in four different aspects: first, in terms of coverage, PyToxo is able to generate penetrance tables of complex epistasis models with realistic heritability and prevalence values; second, in terms of accuracy, its average error is in the order of magnitude of $10^{-17}$, while the maximum error will never exceed the order of $10^{-8}$; third, in terms of speed, PyToxo is very fast, it is able to compute a penetrance table in less than one minute, even for order 8 epistasis models; fourth, in terms of ease of use, PyToxo can be used as a library, through a CLI or through a GUI. This last option will be especially useful for those users not experts in programming or command-line execution environments. PyToxo is cross-platform and it was successfully tested on Linux Ubuntu 20, Microsoft Windows 10 and MacOS Big Sur. In addition, PyToxo is available through the official Python PyPI repository (https://pypi.org/project/pytoxo/) and it can be easily installed  with a single command: pip install pytoxo.

PyToxo is available to the whole scientific community as open-source software. The source code of PyToxo, detailed users guides, and all the models and code examples used in this paper are available in the Github repository: https://github.com/bglezseoane/pytoxo.

## Availability and requirements

Project name: PyToxo

Project home page: https://github.com/bglezseoane/pytoxo

Operating system(s): Platform independent

Programming language: Python

Other requirements: None

License: GPLv3

Any restrictions to use by non-academics: None

## Supplementary Information

**Additional file 1.** Excel document containing additional runtime and precision results from a more extensive examination.

## Data Availability

The source code of Pytoxo, instructions of use, all the models and code examples used in this paper, as well as the results of all the tests executed are available in the Github repository: https://github.com/bglezseoane/pytoxo.

## Abbreviations

CLI: Command-Line Interface
CSV: Comma-Separated Values
GUI: Graphical User Interface
GWAS: Genome-Wide Association Study
MAF: Minor Allele Frequency
SNP: Single Nucleotide Polymorphism
